# En Guard! The Interactions between Adenoviruses and the DNA Damage Response

**DOI:** 10.3390/v12090996

**Published:** 2020-09-07

**Authors:** Tamar Kleinberger

**Affiliations:** Department of Molecular Microbiology, Faculty of Medicine, Technion—Israel Institute of Technology, 1 Efron St., Bat Galim, Haifa 31096, Israel; tamark@technion.ac.il; Tel.: +972-48-295-257

**Keywords:** adenovirus, DNA damage response (DDR), double-strand breaks (DSB), single-strand breaks (SSB), MRN, DNA-PK, PARP-1, E1B-55K, E4orf3, E4orf4, E4orf6, pVII

## Abstract

Virus–host cell interactions include several skirmishes between the virus and its host, and the DNA damage response (DDR) network is one of their important battlegrounds. Although some aspects of the DDR are exploited by adenovirus (Ad) to improve virus replication, especially at the early phase of infection, a large body of evidence demonstrates that Ad devotes many of its proteins, including E1B-55K, E4orf3, E4orf4, E4orf6, and core protein VII, and utilizes varied mechanisms to inhibit the DDR. These findings indicate that the DDR would strongly restrict Ad replication if allowed to function efficiently. Various Ad serotypes inactivate DNA damage sensors, including the Mre11-Rad50-Nbs1 (MRN) complex, DNA-dependent protein kinase (DNA-PK), and Poly (ADP-ribose) polymerase 1 (PARP-1). As a result, these viruses inhibit signaling via DDR transducers, such as the ataxia-telangiectasia mutated (ATM) and ATM- and Rad3-related (ATR) kinases, to downstream effectors. The different Ad serotypes utilize both shared and distinct mechanisms to inhibit various branches of the DDR. The aim of this review is to understand the interactions between Ad proteins and the DDR and to appreciate how these interactions contribute to viral replication.

## 1. The DNA Damage Response

Maintaining genome integrity, both during DNA replication and following exposure to exogenous or endogenous DNA-damaging agents, is crucial for normal cell survival and prevents pathologies such as cancer [1,2]. Many types of DNA lesions may occur, including chemical modification of bases, DNA–protein crosslinks (DPC), DNA double- or single-strand breaks (DSB and SSB, respectively), accumulation of single-stranded DNA (ssDNA), and mismatched DNA bases. The DNA damage response (DDR) network is a cellular system that has evolved to minimize disruption to genome integrity. This network consists of several pathways that detect the various types of DNA damage and initiate signaling cascades that facilitate DNA repair (reviewed in [3,4,5]). The DDR signaling modules include sensors of DNA damage, each of which detects specific DNA lesions, signal transducers, and downstream effectors.

Sensor proteins that were extensively studied include the Mre11-Rad50-Nbs1 (MRN) complex that detects DSBs and stalled replication forks [6,7,8]; Ku proteins that detect DSBs and become part of the DNA-dependent protein kinase (DNA-PK) holoenzyme [9]; replication protein A (RPA) that interacts with ssDNA [10]; and Poly (ADP-ribose) polymerase 1 (PARP-1) as well as other PARP isoforms that can detect SSBs, DSBs, and base modifications [11]. The sensors recruit signal transducers, such as members of the phosphatidylinositol-3-kinase-like protein kinase family (PIKK), including ataxia-telangiectasia mutated (ATM), ATM- and Rad3-related (ATR), and the catalytic subunit of DNA-PK (DNA-PKcs) [3,5]. These transducers activate downstream effectors that amplify the signal, modulate chromatin, and induce cell cycle arrest and DNA repair. Activation of cell cycle checkpoints prevents cell cycle progression until repair is complete. When excessive DNA damage has occurred, cell death may be induced to eliminate cells with highly damaged genomes [1]. DSBs are thought to be the most significant and most harmful type of DNA damage because they can lead to cell death when unrepaired, and if mis-repaired, they can cause genome rearrangements, an early step in carcinogenesis [12]. Two major repair pathways can be utilized to repair DSBs—non-homologous end joining (NHEJ) and homologous recombination (HR). NHEJ predominates during the G1 and G2 phases of the cell cycle, whereas HR peaks in mid-S phase when sister chromatids are present to guide repair by homologous recombination [13].

The MRN sensor consists of three subunits—Mre11, Rad50, and Nbs1. Mre11 binds DNA and has endo- and exonucleolytic activities against single-stranded DNA (ssDNA) and double-stranded DNA (dsDNA) substrates. Rad50 has DNA-binding and unwinding properties, and it has been suggested to play a role in keeping the two DNA ends of the DSB together by dimerization [14]. Nbs1 can translocate the MRN complex to the nucleus by virtue of its nuclear localization signal and its ability to bind the Mre11–Rad50 dimer. Nbs1 also contains an ATM-binding sequence that allows recruitment of this kinase to MRN-bound damage sites to initiate ATM-mediated DNA damage signaling. Furthermore, Nbs1 is involved in binding the phosphorylated form of the histone variant H2AX, a substrate of ATM, allowing the MRN complex to persist at the damage site [15]. In addition to its role in signal initiation and transmission through ATM, the MRN complex also participates directly in the repair process by using its nucleolytic activity for end resection at DSBs. The resulting 3′ ssDNA overhangs on each side of the DSB are used for the HR process of repair. End resection may lead to activation of the ATR kinase by the ssDNA (reviewed in [16]).

Both ATM and ATR can transduce the MRN-generated DNA damage signal, although ATM has been recognized as its main transducer. The precise mechanism of ATM activation is not fully understood. In response to DNA damage, ATM undergoes autophosphorylation on S1981, which was proposed to convert the kinase from an inactive dimer into active monomers. However, other studies contradicted a role for autophosphorylation in ATM activation. ATM is recruited to DSB sites by Nbs1, and the MRN complex was shown to stimulate ATM kinase activity and to enhance ATM signaling (reviewed in [17]). Furthermore, the TIP60/KAT5 acetyltransferase is recruited to damaged chromatin where it is activated, and it then further activates ATM by acetylating it on K3016 [17]. ATM phosphorylates hundreds of substrates, including the highly studied histone variant H2AX (called γH2AX once phosphorylated), Nbs1, BRCA1, Chk2, p53, and others [18,19]. BRCA1 and Rad51 are involved in HR repair of DSBs, whereas Chk2 and p53 activate cell cycle checkpoints. The initial local activation of the DDR through sensors and transducers is amplified globally over several megabases of adjacent chromatin by ATM phosphorylation of H2AX at Ser139 [20]. The scaffolding Mdc1 protein associates with γH2AX and recruits additional MRN, DDR kinases, and other proteins to create DDR foci that facilitate efficient signaling [5].

Ku proteins, in the form of a Ku70/Ku80 heterodimer, rapidly detect DSBs and bind to the damage site within seconds. These proteins keep the two ends of the broken DNA molecule together and recruit the catalytic kinase subunit, DNA-PKcs, to generate the DNA-PK holoenzyme. Following translocation events at the DSB, DNA-PKcs contacts the DSB end and is activated. The active DNA-PK holoenzyme is required for DNA repair by NHEJ, but was also reported to contribute to HR. DNA-PK undergoes autophosphorylation and phosphorylates many substrates, including components of the NHEJ machinery, such as the Ku proteins, DNA ligase IV, the Werner syndrome DNA helicase (WRN), and others. Some of these phosphorylation events have been reported to influence the DSB repair process [9,21].

RPA is a heterotrimer consisting of the RPA70, RPA32, and RPA14 subunits. It binds ssDNA with high affinity and interacts with multiple DNA replication and repair proteins. These interactions facilitate the contribution of RPA to normal DNA replication, and under replication stress conditions it acts as a scaffold for recruitment of checkpoint and fork repair proteins [10]. The RPA70 subunit binds the ATR-interacting protein (ATRIP) to recruit ATR to regions of ssDNA. The ATR-ATRIP complex engages the DNA Topoisomerase II Binding Protein 1 (TOPBP1) through a mediating complex, RAD9-RAD1-HUS1 (9-1-1), which associates with dsDNA junctions near RPA-loaded ssDNA. TOPBP1 then activates the ATR kinase [22,23]. Many proteins that participate in ATR signaling, including RPA, Rad17, TOPBP1, and Claspin, are substrates of ATR. In addition, ATR phosphorylates the checkpoint kinase Chk1 as well as several replication proteins to allow checkpoint activation and promote replication-fork stability and the recovery of stalled forks, thus facilitating the completion of replication [17,22].

ATM, ATR, and DNA-PK share some overlapping substrates, such as γH2AX, but each of them also has independent targets [3].

PARP-1 is a member of a large family of protein isoforms that contain a homologous domain responsible for Poly (ADP-ribose) (PAR) synthesis known as parylation. Some members of this family, including PARP-1, PARP-2, PARP-3, and tankyrase, play a role in DNA damage sensing. PARP-1 is recruited to various types of DNA lesions, including SSBs, DSBs, apurinic/apyrimidinic lesions, and others [24]. PARP-1 binding to DNA lesions results in a local unfolding of an autoinhibitory PARP-1 module, which leads to correct binding of the NAD+ substrate [25,26]. Activation of PARP-1 at the damage site is rapid and results in utilization of NAD+ to generate long PAR chains, including branched chains, which are attached to PARP-1 itself and to many other proteins. Additional mechanisms are also utilized to enhance PARP-1 activation, including interactions with protein partners and various posttranslational modifications [24,27,28]. Once parylation has occurred at the damage site, several proteins containing a PAR-binding region are recruited to the lesion [29]. These proteins continue to transmit the DNA damage signal. After the DDR has been launched, PAR chains are removed by hydrolyzing enzymes such as Poly (ADP-ribose) glycohydrolase (PARG) [30].

## 2. Mechanisms of DDR Induction by Adenoviruses 

Adenoviruses (Ads) are a family of viruses with linear dsDNA genomes in the range of 30–36 Kb, which replicate in the nucleus. They have a wide scope of vertebrate hosts, and there are more than 50 Ad serotypes in humans alone, belonging to seven groups (A–G). The various serotypes infect different tissues and cause several diseases, including respiratory infections, conjunctivitis, and gastrointestinal syndromes [31]. However, despite differences in tissue tropism, all human Ads have similar genome structures and undergo a similar replication cycle [31,32]. Highly studied human Ad serotypes include Ad2/5 (group C), Ad12 (group A), Ads 9 and 36 (group D), and others.

The Ad genome contains five early transcription units (E1A, E1B, E2, E3, and E4), two delayed early units, and one major late unit. These transcription units give rise to numerous mRNAs by alternative splicing and differential polyadenylation. For example, the Ad E4 transcription unit generates at least 18 different mRNAs that encode seven polypeptides from open reading frames (orfs) found in the E4 mRNAs (E4orf1, 2, 3, 3/4, 4, 6, 6/7) [31].

Three viral proteins encoded by the E2 region are required for Ad DNA replication—the preterminal protein (pTP), the Ad polymerase (Ad Pol), and the DNA-binding protein (DBP). DNA replication occurs by a strand displacement mechanism with the help of cellular factors. The Ad pTP protein acts as a primer for replication initiation. Upon encapsidation, this protein is cleaved by the Ad protease to generate the terminal protein (TP), which associates with viral genomes present in mature virions and with the incoming viral genome. The Ad DBP enhances the binding of Ad Pol to the origin of replication and the attachment of the first nucleotide to the pTP. After initiation, the Ad Pol synthesizes one complimentary DNA strand while the non-template strand is displaced and binds DBP. This displaced strand is subsequently replicated to generate a second dsDNA genome; however, the ssDNA intermediates persist in the cells for a while (reviewed in [33]).

Ad genomes could potentially be detected by the DDR system at several stages of infection. The incoming viral genome, as well as the replicating genomes, have ends that are similar to DSBs and could be recognized by DSB sensors. However, viral DNA ends are bound by the Ad pTP or TP, which could partially protect them. Replication intermediates include large amounts of ssDNA, which could potentially trigger ATR activation. However, the ssDNA molecules are coated and protected by the Ad DBP. Another proposed mechanism for triggering the DDR involves recognition of the Ad TP bound covalently to the 5′ end of the genome as a DNA–protein adduct that could trigger removal of TP by a mechanism of DNA–protein crosslink repair (DPC) [34], resulting in inhibition of viral replication [35]. Indeed, during infection with an E4-deleted Ad mutant, the Ad TP was removed from the ends of the viral DNA in a manner similar to the removal of end-blocking proteins from cellular DSBs [36]. This process required the CTBP-Interacting Protein (CtIP) and the endonucleolytic activity of Mre11, and the protein-linked oligonucleotides that were excised from the viral genome were similar in size to the oligonucleotides that remain attached to Top2 and Spo11 after they have been removed from the 5′ termini of DSBs during etoposide chemotherapy and meiotic recombination, respectively. Thus, the ends of the Ad genome can become exposed and be recognized by the DDR. One of the first reports proposing that Ad genomes were targeted by the DDR showed that infection with Ad mutants lacking the E4 region resulted in ligation of viral genomes to generate concatemers [37]. Sequence analysis of end-to-end junctions in concatemers revealed large heterogeneous deletions at the junctions and indicated that genomes were ligated through a process involving NHEJ. However, as cleavage of the Ad TP protein and degradation of Ad genome termini were not detected early during infection, concatenation was proposed to be a late event [38]. In contrast to mutant Ads, Wild-Type (WT) Ad genomes do not undergo concatenation, suggesting the presence of Ad proteins that inhibit the DDR. A detailed report of the effects of Ads on the DDR is presented below.

In addition to identification of Ad genomes as DNA damage by the DDR, the virus can also induce cellular DNA damage. Initially, Ad12 was reported to induce breaks in chromosomes 1 and 17 in human embryo kidney cells [39]. It was later shown that non-random, site-specific chromosome damage was induced by additional group A Ads, including serotypes 18 and 31 [40]. Upon further studies, it was found that the Ad E1A protein sensitized cells to toxicity induced by DNA-damaging agents and promoted aberrant cellular DNA replication, leading to replicative stress in a cMyc-dependent manner [41,42,43]. Unresolved replicative stress could contribute to genomic instability. Modulation of p53 function by many types of Ad could also affect host genome stability [44]. At the early stages of infection, the MRN DSB sensor binds viral genomes and activates a local ATM-dependent anti-viral response that would inhibit viral replication in the absence of MRN-inactivating Ad proteins without interfering with cellular DNA replication. However, at later stages, Ad replication triggers an MRN-independent global ATM signaling that does not affect viral replication and is associated with global γH2AX activation [45,46]. It is possible that global H2AX phosphorylation facilitates recruitment of repair proteins to γH2AX, away from viral replication centers (RCs) [46]. It was also proposed that this global DDR activation may have a role in modulation of the host immune response to virus infection [45]. During infection, Ad genomes themselves are quite stable and do not accumulate mutations at high rates, even when PIKK kinases are inhibited [47,48].

In the following sections, “Ad” refers to Ad2/5, and other serotypes are specifically identified.

## 3. Exploitation vs. Inhibition of the DDR by Adenoviruses

Many DNA viruses were reported to exploit components of the DDR network to enhance efficient replication of the viral genome. Examples include polyoma [49,50,51] and papilloma [52,53,54,55] viruses, which activate ATM, ATR, and other DDR proteins to support their DNA replication by preserving viral replication fork integrity and recruiting repair factors to replicate viral DNA. Various herpesviruses also utilize components of the DDR machinery to support diverse aspects of their replication (reviewed in [56,57,58]). However, in many cases, the DDR acts as an anti-viral defense mechanism that restricts viral replication, and many viruses have evolved numerous mechanisms to inactivate this process in order to enhance the efficiency of their replication. Ad is frequently used as an example of a DNA virus that evolved multiple mechanisms to limit the DDR, and these mechanisms will be described in Section 4 and Section 5. However, there are also a few reports suggesting certain benefits of DDR activity to the virus.

During Ad replication, some DDR proteins are recruited to viral RCs, including RPA32, ATR, ATRIP, Rad9, TOPBP1, Rad17, hnRNPUL1 (also known as E1B-AP5), DNA-PK, and SLX4 [59,60,61,62,63,64]. These proteins may be drafted to advance viral processes. For example, SLX4 was shown to promote Ad5 genome accumulation and protein production [63]; DNA-PK was shown to be activated early during infection with an Ad5 mutant lacking the whole E4 region except E4orf4, and to facilitate E4orf4-induced inactivation of ATM and ATR signaling at the early phase [62]; and ATR activation by Ad12 and hyperphosphorylation of RPA32 were suggested to contribute to inhibition of cellular DNA replication for efficient viral replication, as RPA hyperphosphorylation was reported to inhibit host DNA replication [59]. In addition, it was proposed that initial nucleation of Ad RCs may be facilitated by DDR proteins that are involved in formation of DNA damage foci on the Ad genome before the onset of synthesis of early viral proteins that inhibit the DDR [65]. This yet unproven hypothesis is supported by the finding that DDR sensors and effectors such as Mre11, Mdc1, and ATM colocalized with viral genomes early during infection [45,66,67]. Some of these proteins were relocalized to other sites or were degraded once early viral proteins that inhibit the DDR accumulated (see Section 5). Additionally, Ad35 DNA replication was reported to increase when hypomorphic Nbs1 cells were reconstituted with Nbs1, suggesting that Ad35 may, at some stage, exploit components of the MRN complex to benefit viral replication. Similarly, Ad12 replication was slightly diminished in cells treated with an ATM inhibitor or in cells lacking ATM [68].

Another DDR pathway that is activated by Ad5 infection and benefits virus replication is the Fanconi anemia (FANC) pathway [69]. This pathway consists of several proteins (FA proteins) that assist other DNA repair proteins to repair DNA inter-strand cross-links. During normal DNA replication or in response to DNA damage, eight of the FANC proteins form a core FANC complex that mediates monoubiquitination of FANCD2 and FANCI [70]. Following monoubiquitination, the FANCD2/FANCI dimer recruits FANCD2 and FANCI Associated Nuclease 1 (FAN1) and relocalizes to nuclear foci containing proteins implicated in the recombination-dependent repair of stalled replication forks and DSBs to assist in DNA repair. Cumulatively, FA proteins play a central role in alleviating replication stress by suppressing dormant origin firing, promoting replication fork stability, and stabilizing common fragile sites. Ad5 was reported to induce FANC core-dependent FANCD2 monoubiquitination and to facilitate its recruitment to viral RCs independently of DDR coordinated by phosphorylated Nbs1 and Chk proteins. FANCD2 monoubiquitination required Ad5 replication. The absence of FANCD2 impaired adenoviral replication and viral genome recombination, suggesting that the FANC branch of the DDR can be exploited by Ad5 for the benefit of the virus [69].

## 4. The Incoming Viral Genome and the DDR

Several reports in the literature addressed the question of whether the incoming viral genome was sufficient to activate the DDR or whether viral DNA replication was required for this process. It has been reported that upon infection with either WT or an E4-deleted Ad, Mdc1, an early participant in irradiation-induced DDR, accumulated in foci before synthesis of the Ad DBP protein was detected [71]. This observation suggested that DDR was activated prior to DNA replication. Single-cell analysis by immunofluorescence further indicated that the MRN complex was degraded and ATM was activated prior to the onset of DNA replication, indicating that input Ad genomes were sufficient to induce the DDR [38]. However, the DNA damage response to the viral genome was attenuated early in infection with Ad mutants lacking both E4orf3 and E4orf6 or E4orf3 and E1B-55K, even after early viral gene expression reached its peak and at the onset of Ad DNA replication [38]. These results suggest the possibility that the incoming viral genome may be protected from the DDR by a mechanism independent of E4orf3, E4orf6, and E1B-55K.

The Ad core protein VII is a basic protein that binds the Ad genome in a histone-like fashion. Its association with the viral genome is observed within virions and during the early phase of infection [72,73,74,75]. Removal of protein VII from the viral genome is correlated with initiation of transcription [76,77,78]. It has been demonstrated that as long as the incoming Ad genome was bound by core protein VII, it was protected from DDR-induced checkpoint signaling reflected by formation of pATM-containing foci [79]. When viral transcription was initiated, core protein VII was released from the viral genome and the DDR was activated [72,79]. It is possible that protein VII masks the incoming viral genome termini and prevents DDR activation until early viral transcription is initiated and early viral proteins that inhibit DDR activation are generated. During Ad infection, protein VII also associates with the host chromatin [80] and inhibits irradiation-induced DDR signaling [81]. Protein VII binds the SET/TAF-1 protein, an oncoprotein that was shown to mediate heterochromatin formation at DSBs via recruitment of factors such as HP1 and KAP1. SET/TAF-1 was also shown to localize to incoming adenovirus genomes and to facilitate deposition of histones on these genomes to promote transcription [82,83,84,85]. Knockdown of SET/TAF-1 partially rescued inhibition of irradiation-induced DDR signaling at the host chromatin by protein VII, suggesting that SET/TAF-1 was required for this inhibition. Moreover, knockdown of SET/TAF-1 also allowed pATM to localize to incoming protein VII-bound viral genomes of mutant Ads lacking the E4 region, indicating that SET/TAF-1 assisted protein VII-facilitated evasion of the DDR by Ad [81].

The chromatin-associated factor and epigenetic reader, SPOC1 (survival-time associated PHD protein in ovarian cancer 1/PHF13), is recruited to DSBs and plays a role in the DDR [86]. SPOC1 was shown to associate with the Ad genome at RCs and repress viral gene expression. Protein VII binds SPOC1 and possibly protects the Ad genome from SPOC1 effects on the Ad chromatin [87]. It is not clear, however, whether this protection is also relevant to the viral-induced DDR process.

## 5. Targeting DNA Damage Sensors and DDR Signaling by Ads

**Figure 1:**
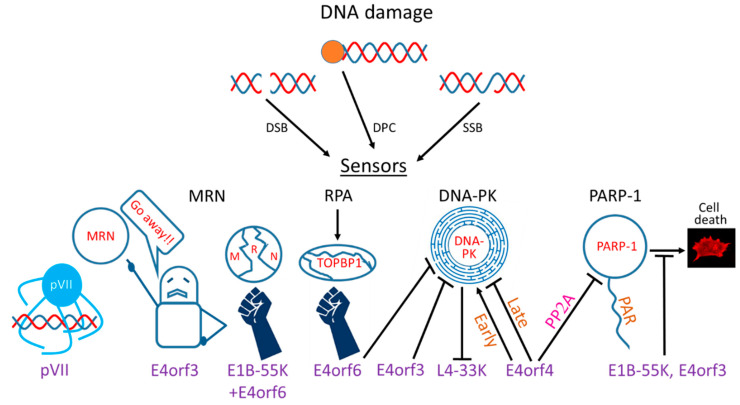
Interactions of adenovirus (Ad) proteins with DNA damage response (DDR) system components. Three types of DNA damage related to Ad infection are shown (DSB: double-strand breaks, SSB: single-strand breaks, DPC: DNA–protein crosslinks). The effects of several Ad proteins involved in the interaction with the DDR are shown in cartoon form. pVII: protein VII.

Ads evolved multiple mechanisms to restrict the DDR by targeting various DDR sensors, transducers, and effectors (Figure 1). However, not all Ad serotypes utilize the same mechanisms to achieve this common goal. Below, the various mechanisms employed by different Ad serotypes to limit the DDR are described (also summarized in Table 1).

### 5.1. Degradation of MRN Subunits 

The MRN complex is a major Ad target that is inactivated by various early Ad proteins through at least two mechanisms—protein degradation and removal from Ad RCs to nuclear tracks and perinuclear aggresomes (see also Section 5.2).

The viral early proteins E1B-55K, a product of the E1 region, and E4orf6, a product of the E4 region, associate jointly with cellular proteins of the Cullin (Cul) family and with Ring-box 1 (Rbx1) and Elongins B and C to form an E3 ubiquitin ligase that is required for the degradation of several host proteins [90,106,107,108]. The E4orf6 protein is responsible for recruiting the cellular E3 ubiquitin ligase components [107,108], whereas the E1B-55K protein is thought to dictate the substrate specificity of the ligase [91,107,108]. Several substrates of the E1B-55K/E4orf6 complex were degraded independently of each other, and mutation analysis of the E1B-55K protein revealed that distinct regions of this protein were required for binding different substrates [91]. The E4orf6 protein contains conserved BC box motifs that resemble sequences in other Elongin-C-interacting proteins, and these motifs were shown to contribute to the formation of an active E3 ubiquitin ligase [109,110]. Although E1B-55K/E4orf6 complexes of all Ad serotypes recruit Cullin-containing E3 ubiquitin ligases, the complexes differ in their composition and function. Thus, Cul5 is present in complexes of serotypes 34, 5, 9, and 4 (representative of groups B, C, D, and E), Cul2 is recruited by serotypes 12 and 40 (representative of groups A and F), and the Ad16 complex binds both Cullins. Furthermore, substrate specificity also differs in the various Ad serotypes. All serotypes tested degraded DNA ligase IV, whereas complexes from some serotypes did not degrade Mre11, p53, or integrin α3 [88,89,104]. However, certain serotypes that did not degrade Mre11 could degrade other MRN components [90,91], and those that could not degrade any of the MRN proteins could mislocalize the complex [68]. These findings demonstrate that inactivation of DNA ligase IV and, hence, of NHEJ as well as of MRN is shared by all Ad groups, and therefore must be crucial for the Ad life cycle. Indeed, it was shown that MRN inhibits Ad replication in the absence of Ad proteins that inactivate it [71,105]. Inactivation of the DDR at various other junctions may be differentially important for the diverse Ads or, alternatively, different components of the same pathway can be targeted by varying Ad groups to achieve the same goal of pathway inactivation. For example, out of representatives of several Ad groups that were tested, only Ad12 caused degradation of the ATR activator TOPBP1. Despite that, other Ad serotypes, such as Ad5 and Ad9 also inhibited ATR activation, as monitored by phosphorylation of the ATR substrate Chk1, and Ad3 and Ad11, initially activated ATR, but later diminished its activation [89]. In the case of Ad5, ATR inactivation was mediated by mislocalization of the MRN complex rather than by TOPBP1 degradation [60]. These results further confirm that different mechanisms can be utilized by various Ad serotypes to inhibit the same DDR branch. Interestingly, Ad serotypes differed in their susceptibility to inhibition by the MRN complex. The replication of Ad5, Ad2, and Ad4 was not affected by the presence of MRN likely because they could inactivate this complex, but Ad35 replication was enhanced in its presence. In contrast, despite the finding that Ad9 and Ad12 targeted MRN either by mislocalization or by degradation, their replication was impaired in its presence, suggesting that targeting MRN by these viruses was not enough to overcome inhibition of viral DNA replication by this complex [68]. Such results are puzzling because MRN is a ubiquitous target of all human Ad groups, and Ads deploy quite a few of their proteins to counteract MRN. However, as suggested by Weitzman and colleagues [68], it is possible that Ad9 and Ad12, which cause conjunctivitis and gastrointestinal disorders, respectively, are able to evade MRN inhibition in conjunctival or gastrointestinal cells, but not in non-physiological cells utilized in the published studies. This is consistent with a related report proposing that the species-specific interaction between Nbs1 and herpes simplex virus 1 is linked to species tropism [111]. MRN also impairs replication of mutant Ad5 (subgroup C) and Ad4 (subgroup E) that lost their ability to inactivate MRN [71,105,112].

The full details of the mechanisms by which MRN inhibits Ad replication may not be fully recognized to date. In addition to the consequences of DDR activation by MRN in response to Ad replication, it has been proposed that MRN binding near viral replication origins located at the ends of the viral genome may physically interfere with the progression of viral DNA replication. This suggestion was based on the finding that, whereas knockdown of MRN components increased the replication of an Ad5 mutant lacking MRN-inactivating proteins (an E1B-55K/E4orf3 double mutant), addition of mirin, an inhibitor of Mre11 nuclease activity, did not have a similar effect. Furthermore, an inhibitor of the MRN signal transducer ATM was less efficient in rescuing mutant Ad replication in comparison with MRN knockdown [45]. However, these results could also be consistent with the reported finding that ATM activation by MRN is nuclease-independent [113] and may suffice in the response to viral replication under some circumstances; in addition, ATR activation may rescue some effects of ATM inhibition.

### 5.2. Degradation of Additional Host Proteins

In addition to MRN, many other proteins that are involved in the DDR as part of their roles in the cell were identified as degradation targets of various Ad serotypes. The first target of this type that was investigated was p53, which was shown to be destined for proteasome-dependent degradation by the Ad5 E1B-55K and E4orf6 proteins [114,115]. As described for Mre11, these proteins recruited an E3 ubiquitin ligase containing Elongins B and C, Cul5, and Rbx-1 to mediate p53 degradation [107,108]. It was later shown that not all Ad serotypes targeted p53 for degradation, and this tumor suppressor accumulated during infection with some Ad serotypes, although not in an active form [89].

In addition to proteins that are involved in DNA damage signaling, such as MRN subunits, TOPBP1, and p53, proteins that are involved directly in DNA repair, such as DNA ligase IV [106,116] and the Bloom helicase (BLM) [97], are also degraded during infection with various Ad serotypes. A contribution of DNA ligase IV to inhibition of Ad replication was not tested directly, but the observation that all Ad serotypes examined degraded this protein, strongly suggested that it restricts Ad replication [89]. Indeed, degradation of DNA ligase IV, which participates in NHEJ, may lead to inhibition of concatenation during infection with all Ad serotypes that were investigated. Knockdown of the second DNA repair enzyme targeted by Ad, BLM, did not affect Ad replication, but it was proposed that other helicases may provide redundant functions to replace BLM. BLM was reported to be involved in resection of DNA breaks, and it is possible that it collaborates with MRN to modify the ends of viral genomes and, therefore, must be disabled. The degradation of both DNA ligase IV and BLM is mediated by the E1B-55K/E4orf6 complex [97,106].

Several other proteins that were reported to play a role in regulation of DNA damage signaling have been identified as Ad degradation targets, although direct effects of their degradation on the Ad response to the DDR have not been established. SMARCAL1 (SWI/SNF Related, Matrix Associated, Actin Dependent Regulator of Chromatin, Subfamily A Like 1) is a DNA-dependent ATPase and ATP-dependent annealing helicase that participates in the response to different types of DNA damage. SMARCAL1 is recruited to ssDNA generated during DSB resection in an RPA-dependent manner, and it both stabilizes replication forks and restores fork integrity. This protein also contributes to DNA replication fork integrity during replication stress [117,118,119]. Both Ad5 and Ad12 reduced SMARCAL1 protein levels in an E1B-55K/E4orf6- and Cul5-dependent manner. Early during infection, SMARCAL1 was recruited to viral RCs through its interaction with RPA, and this recruitment was also regulated by ATR- and cyclin-dependent kinase (CDK)-dependent phosphorylation. The degradation of SMARCAL1 in Ad-infected cells led to attenuation of cellular DNA replication, but no direct effect on improving viral replication has been shown yet [96].

The tankyrase 1 binding protein 1 (TNKS1BP1, also known as Tab182) is an ATM and ATR substrate that is phosphorylated in response to ionizing radiation (IR) [19]. It functions in the HR pathway of DSB repair by facilitating PARP-1-dependent autophosphorylation of DNA-PKcs [120,121]. TNKS1BP1 is a component of the CCR4-NOT Transcription Complex (CNOT), which is engaged in transcriptional regulation, deadenylation, and E3 ubiquitin ligase activity. TNKS1BP1 is degraded during infection with Ad5 and 12, but remains stable upon infection with the Ad serotypes 4, 7, 9, and 11. Some of the other CNOT subunits, but not all of them, are also depleted during infection with Ad5 and 12. When TNKS1BP1, and to a larger extent CNOT1, were depleted by siRNA, the expression of E1A and levels of viral DNA were enhanced. Surprisingly, however, CNOT1 remained stable during Ad infection. Thus, some CNOT subunits are targeted for E1B-55K/E4orf6-mediated proteasomal degradation by certain Ad serotypes, achieving enhanced E1A expression [95]. However, direct effects of CNOT complex subunits on DDR activation during Ad infection have not been addressed yet.

SPOC1 has been identified as a regulator of DDR and chromatin structure [86,122]. SPOC1 was reported to associate with the Ad5 core protein VII, to accumulate at viral RCs, and to be targeted for proteasomal degradation by the Ad5 E1B-55K/E4orf6 complex. SPOC1 restricted Ad replication, as its overexpression reduced Ad gene expression and DNA accumulation, whereas its knockdown produced the opposite effects. Depletion of SPOC1 led to release of several factors and elimination of repressive histone marks from chromatin, resulting in chromatin decondensation that facilitated access of regulators to the DNA. Prior to its degradation, SPOC1 affected viral gene expression by binding Ad promoters and impacting transcription [87]. Interestingly, another chromatin remodeler, the acetyltransferase TIP60, was also degraded during Ad5 infection [94]. It was reported that TIP60 binding to H3K9me3 at DSBs stimulated its enzymatic activity and facilitated efficient ATM activation by acetylation [123]. It is possible, therefore, that TIP60 depletion during Ad infection contributes to inhibition of ATM signaling by the virus. However, in the context of Ad infection, studies that have been reported to date presented evidence only for repression of Ad gene expression by TIP60, similarly to SPOC1. 

Additional Ad degradation targets that are not known to participate in the DDR have also been described, including TIF1γ [124], integrin α3 [125], and DAXX [126].

The degradation of most Ad targets described above was mediated by the E1B-55K/E4orf6 complex. However, a minority of Ad degradation substrates were targeted for degradation otherwise. Degradation of TOPBP1 by Ad12 required only the Ad12 E4orf6 protein, as it appeared that unlike E4orf6 proteins of other Ad serotypes, this protein could bind both the Cullin-based E3 ubiquitin ligase and the TOPBP1 substrate [104]. In another variation, DAXX degradation was facilitated by the Ad5 E1B-55K protein only, although it was not clear why E4orf6 was dispensable for degradation of this specific substrate, whereas it was required for E3 ubiquitin ligase recruitment to other substrates [126]. In contrast to all other known Ad degradation targets, the transcriptional intermediary factor 1γ (TIF1γ) was targeted for proteasomal degradation by the Ad5 E4orf3 protein, but not by E1B-55K, E4orf6, or both, in a Cullin-independent manner. These observations suggest that E4orf3 utilizes a different cellular ubiquitin ligase to promote TIF1γ degradation [124].

### 5.3. Relocalization of MRN and Other DDR Proteins

In addition to degradation of MRN subunits, certain Ad serotypes employ another mechanism to prevent MRN-dependent activation of the DDR at viral RCs through relocalization of the complex to other cellular sites by the Ad E4orf3 protein [64,112].

Promyelocytic leukemia nuclear bodies (PML-NBs, also known as nuclear domain 10: ND10) are multiprotein complexes that are detected in nuclei as punctate structures. These entities have been reported to participate in several cellular processes, including transcriptional regulation, induction of apoptosis, DNA damage repair, protein modification, and antiviral responses including activation of the interferon system [127]. To fulfill their role in DNA damage sensing they contain some DDR proteins [128]. During Ad infection, a multimeric form of E4orf3 reorganizes the punctate PML-NBs into ‘track-like’ structures [129,130,131,132,133]. The disruption of PML-NBs counteracts their contribution to inhibition of Ad replication. Restriction of PML-NB anti-viral activity is facilitated by directly repressing the antiviral function of individual PML-NB components and by inhibition of the interferon response [134,135,136]. Furthermore, E4orf3 of Ads 2 and 5 mislocalizes the MRN complex, which, instead of accumulating in viral RCs, is sequestered in PML-containing tracks where it is unable to direct a DNA damage response to viral DNA [90,112]. Mislocalization of MRN by Ad5 was specifically proposed to inhibit ATR activation [60]. On the other hand, E4orf3 proteins of Ad12 failed to exclude the MRN complex from viral RCs prior to Mre11 degradation. They were also unable to rescue defects involving concatemer formation and diminished late protein production of a virus with a deletion of E4 [64]. Further studies revealed additional Ad serotypes that were unable to mislocalize MRN, including Ad3, 7, 11, 35 [68,89]. Inconsistent results were presented regarding Ads 4 and 9 which were shown to mislocalize MRN in some studies [68,89] but not in others [64,99]. Altogether, the results indicate that MRN mislocalization to nuclear tracks by E4orf3 is not a conserved function in all human Ad groups.

In addition to MRN inhibition, E4orf3 also suppresses the DDR downstream effector p53 by epigenetic silencing of its target genes [100], and recruits additional host proteins, such as transcription factors TIF1α, TIF1γ, and TFII-I to nuclear tracks, where they may be rendered inactive [35].

Conjugation of members of the small ubiquitin-related modifier (SUMO) family of proteins contributes to regulation of several cellular processes, including transcription, replication, chromosome segregation, and DNA repair [137,138]. During Ad infection, E4orf3 relocalizes SUMO proteins to E4orf3 nuclear tracks, and sumoylation of several host proteins is increased. Most of the E4orf3 SUMO targets, including the MRN subunits Mre11 and Nbs1, participate in DNA damage signaling and repair processes [99,139]. E4orf3-mediated sumoylation occurs only once the target proteins localize to E4orf3 nuclear tracks. Both generation of nuclear tracks and SUMO recruitment are shared by E4orf3 proteins encoded by Ad serotypes belonging to groups A–E [99]. However, whereas TIF-1γ, for example, is mislocalized and sumoylated by E4orf3 proteins of all five Ad serotypes tested, Ad serotypes that do not cause MRN mislocalization also fail to cause MRN sumoylation [99,140]. Once sumoylated, some of the E4orf3 targets are destined for degradation by the ubiquitin-proteasome pathway, while others, like Mre11 and Nbs1, are not degraded as a result of E4orf3-induced sumoylation, and the impact of this modification on their function remains unclear (reviewed in [35]).

In addition to the formation of nuclear tracks, Ad infection also induces formation of cytoplasmic perinuclear aggresomes [92,93]. These structures contain misfolded and aggregated proteins that have been removed and transported by dynein along microtubules towards the microtubule-organizing center [141]. Aggresomes are cleared by aggrephagy, a selective autophagic clearance mechanism. This process may play a cytoprotective role in response to the accumulation of aggregates containing misfolded proteins when other mechanisms to eliminate them are impaired or overwhelmed [142]. The Ad5 E1B-55K protein has been reported to localize to aggresomes in Ad E1-transformed cells and during infection [92,93,143]. It appears that during infection with Ad5, MRN is first localized to the nuclear tracks, where it binds E1B-55K, and later, either E4orf3 or E4orf6 as well as E1B-55K are required for E1B-55K aggresome formation, and Mre11 is exported to the aggresomes. It has also been shown that nuclear export of MRN complexes to E1B-55K aggresomes increases the rate of their proteasomal degradation. Thus, the export of MRN to aggresomes serves to sequester this complex and prevent its detrimental effect on Ad replication as well as to facilitate its efficient degradation. Indeed, aggresomes accumulate proteasomes, which could contribute to rapid substrate degradation [92,93]. It should be noted that, similarly to the relocalization of MRN to PML tracks, infection with some Ad serotypes did not result in MRN mislocalization to E1B-55K aggresomes [64,144], indicating that a variety of mechanisms are utilized by different Ads to inhibit MRN and the DDR. As a result of both degradation and mislocalization, DDR proteins, such as MRN components, are under-represented in proteomes associated with Ad genomes during infection [63].

### 5.4. Ad Effects on the ATM and ATR Transducers of MRN Signaling

ATM is recruited to DSBs by MRN and transduces DDR signaling. Ad5 was found to suppress ATM activation in viral RCs, and further ATM inhibition by other means did not significantly affect WT Ad5 replication. However, reducing ATM activity enhanced E4-mutant Ad5 replication, suggesting that ATM activity was required to inhibit it [45,66]. In contrast, many other WT Ad serotypes, including Ad2, Ad4, Ad9, Ad12, and Ad35, manifested accumulation of activated ATM in their RCs [68], although the impact of ATM activation in these cases has not been investigated. Because most of these Ad serotypes target and inactivate the MRN complex, it is possible that ATM activation is mediated by residual MRN left in the RCs. Alternatively, because ATM activation occurs very close to viral RCs [45], and based on the finding that ATM can be activated by disruption of cellular chromatin [145], it was proposed that the assembly of viral genomes in nuclear RCs may lead to disruption of the surrounding chromatin, thus activating ATM [45]. Experiments with mutant Ad5 viruses suggested that pATM must be physically recruited to viral DNA to mediate its inhibitory effect on replication [45,146]. ATM did not impair replication of Ad9 and Ad12, which were attenuated by MRN, indicating that inhibition of viral DNA replication by MRN was unlikely to be mediated by ATM activation during these infections [68]. ATM activation by all Ad serotypes tested, including Ad5, was also shown by monitoring increased phosphorylation of Kap1, an ATM substrate [89], which, in the case of Ad5, could represent the global ATM activation, which does not inhibit Ad5 replication [45].

ATR activation is suppressed following MRN inactivation during Ad5 infection [60,61,89]. During infection with WT Ad5, ATR is recruited to viral RCs but is not activated, as determined by the low levels of Chk1 phosphorylation. However, when the cells are infected with an E4-mutant virus, MRN is recruited to RCs and facilitates robust ATR activation. This was demonstrated by comparing ATR activation by the mutant Ad in cells lacking MRN components and upon reconstitution with WT MRN proteins. The ATR activation mediated by MRN does not require the Mre11 nuclease activity or ATM activation, and ATR recruitment to Ad RCs does not require MRN. Furthermore, MRN mislocalization by E4orf3 is responsible for inhibition of ATR signaling in Ad5-infected cells, and E4orf3 was shown to reduce MRN mobility in the nucleus, possibly by sequestration in nuclear tracks, as seen by analysis of recovery after bleaching of fluorescently tagged proteins [60,61]. As described above (Section 5.1), Ad12 has adopted a different mechanism to inhibit ATR signaling by degrading the ATR activator TOPBP1. No other Ad serotypes that were tested utilized this mechanism to inhibit ATR signaling [104].

During infection with Ad3, Ad4, Ad7, and Ad11, ATR was activated, resulting in phosphorylation of Chk1. Despite this, virus replication continued. It appears that at least for Ad5, Ad12, and Ad9, the inhibition of ATR signaling is beneficial, and occurs by Ad12-induced degradation of TOPBP1, Ad5-induced inhibition of ATR signaling by degradation and/or mislocalization of Mre11, and Ad9-induced MRN complex relocalization to PML-containing nuclear tracks [89].

The Ad5 E4orf4 protein was also shown to inhibit the ATM and ATR signaling pathways that were activated during infection with an E4-mutant Ad, and it required its major partner, PP2A, for this function [102]. When E4orf4 was expressed alone, attenuation of ATM and ATR resulted in accumulation of DNA damage. Inhibition of the ATM pathway by E4orf4 was independent of ATR inhibition and vice versa, and both inhibition events were shown to contribute to the efficiency of Ad infection. ATM attenuation increased the levels of mutant Ad DNA and late protein expression, whereas obstruction of ATR enhanced late gene expression only [102]. The effects of ATR on expression of late Ad genes may possibly result from ATR effects on alternative splicing [147], which plays an important role in regulation of late gene expression. Some reports demonstrated that, whereas ATM interfered with DNA replication and late protein expression of an E4-mutant Ad, ATR had no effect [45,66]. However, the intensity of ATR effects could vary in different cell lines and they are most apparent when ATM is absent as well [102].

### 5.5. Targeting DNA-PK

DNA-PK is targeted by several Ad proteins, suggesting that its modulation is highly important to the virus. The Ad E4orf3 and E4orf6 proteins associate with DNA-PK, and E4orf6 was shown to inhibit V(D)J recombination, which is normally regulated by this kinase. Moreover, genome concatenation in E4 mutant-infected cells required DNA PK, whereas E4orf3 and E4orf6 could each inhibit concatemer formation. It was concluded that these E4 proteins prevent genome concatenation by binding DNA-PK and inhibiting its ability to induce repair by NHEJ [37,101]. Expression of E4orf6 alone did not inhibit the DNA-PKcs kinase activity toward heterologous substrates, but prolonged autophosphorylation of DNA-PKcs at Thr-2609. It also prolonged H2AX phosphorylation in a PP2A-dependent manner and sensitized cells to damage-induced toxicity [148]. The prolonged DNA damage signaling in the presence of E4orf6 resulted in cell death [149].

Another Ad protein, L4-33K, was reported to bind the DNA-PK catalytic subunit [150]. This protein is an Ad alternative splicing regulator that activates a shift from early accumulation of the L1-52,55K RNA to the late generation of the L1-IIIa mRNA [151]. The L4-33K protein was shown to be highly phosphorylated by DNA-PK in vitro, and production of the late-accumulating L1-IIIa mRNA was enhanced in cells lacking DNA-PKcs [150]. The observation that L4-33K phosphorylation by DNA-PK did not require activation by dsDNA supported a DDR-independent consequence for the interaction between the two proteins. The results indicate that DNA-PK inhibits the temporal switch in L1 alternative RNA splicing, an effect that would be required at the early stage of infection when the L1-52,55K RNA must accumulate.

A fourth Ad protein that was reported to bind DNA-PK is E4orf4, and the complex contained both DNA-PKcs and Ku70 [62]. When the E4orf4 effect on DNA-PK activation was examined, it was shown that whereas an E4-mutant Ad stimulated DNA-PK autophosphorylation, the presence of E4orf4 in the mutant background had a biphasic effect on DNA-PK activation. Early during infection, E4orf4 activated DNA-PK, whereas it later inhibited its activity as monitored by autophosphorylation levels of the kinase. Interestingly, DNA-PK activation was shown to be required for the ability of E4orf4 to reduce ATM and ATR signaling. Consequently, although inhibition of DNA-PK improved the replication of an E4-mutant Ad even when exerted early during infection, replication of an E4orf4-expressing E4-mutant Ad was improved further when DNA-PK was inhibited only later during infection, allowing E4orf4 to function in collaboration with DNA-PK at the early stage. Furthermore, DNA-PK was recruited to Ad RCs early in infection, but colocalization with the RCs was reduced late in infection [62]. The results clearly demonstrated that DNA-PK activity was beneficial for Ad replication early in infection, but became detrimental to the virus in later infection stages, and was then inhibited by E4orf4. Inhibition by E4orf3 and E4orf6 may also occur late in infection, as the requirement to inhibit genome concatenation is important at the late stages of the viral life cycle [38]. Thus, DNA-PK activation by E4orf4 contributed to modulation of ATM and ATR signaling, and inhibition of DNA-PK late during Ad infection did not only impact the DDR and prevented genome concatenation [101], but also improved the shift from early to late L1 RNA splicing events [150] and led to a pronounced increase in expression of an Ad cement protein, pIX [62]. This viral protein contributes to stabilization of the hexon shell and further improves virus infectivity [152].

In addition to interactions between Ad proteins and DNA-PKcs, the Ad E1A protein was reported to bind Ku70 [153]. Surprisingly, knock down of Ku70 by siRNA reduced virus titer three-fold, suggesting that the Ku70 protein was required to enhance viral replication, in apparent contradiction to the results described above showing that DNA-PK inhibition enhanced Ad replication. Furthermore, Ku70 knockdown did not result in reduction of viral gene expression and protein levels or viral genome replication. However, the virus could not efficiently induce S-phase in Ku70-depleted cells and upregulated expression of the cell cycle inhibitor p21. Ku70 also appeared to bind within gene bodies in viral genomes. It was suggested that Ku70 may play a role in regulation of cell cycle gene expression [153] and thus affect Ad replication indirectly. Still, mounting evidence strongly suggests that the DNA-PK holoenzyme and its activation during Ad infection must be inhibited as infection progresses.

### 5.6. Targeting PARP-1

In addition to binding DNA-PK, the Ad E4orf4 protein also associates with PARP-1 [103]. This association requires the activities of at least two E4orf4 partners: protein phosphatase 2A (PP2A) and PARP itself. PARP-1 is recruited to the vicinity of Ad RCs, but recruitment does not require either E4orf4, which is also present in RCs, or PARP activity. E4orf4 was shown to inhibit parylation that was stimulated both by chemically induced DNA damage and by Ad infection, and this inhibition required the interaction of E4orf4 with its major partner, PP2A [103]. Indeed, PARP-1 appeared to be hypophosphorylated in the presence of E4orf4, and this state of reduced phosphorylation likely contributed to PARP-1’s diminished activity, as phosphorylation of various residues was shown to enhance parylation by PARP-1 [154,155]. Reducing PARP activity elevated the ability of E4orf4 to diminish ATM and ATR signaling, but did not decrease their activation in the absence of E4orf4, suggesting that PARP inhibition plays a role in attenuation of these DDR branches by E4orf4. Thus, restraining PARP can contribute to the Ad life cycle both by allowing E4orf4 to inhibit ATM and ATR signaling, which contributes to the efficiency of Ad replication [102], and by inhibiting additional PARP-regulated processes. Indeed, attenuation of PARP enhanced the efficiency of replication of an E4-mutant Ad [103], which is normally deficient in replication, at least in part because of its inability to inhibit several DDR branches.

The contribution of PARP inhibition to Ad replication was demonstrated in an additional report showing that Ad genome replication stimulated parylation of both cellular and late viral proteins, and that two Ad proteins, E1B-55K and E4orf3, inhibited one of the consequences of this parylation, namely nuclear fragmentation [98]. The utilization of several Ad proteins to counteract PARP activation reinforces the importance of diminishing PARP activity to the Ad life cycle. It was suggested that accumulation of the PAR polymer, which is synthesized by PARP, acts as a cell-death-inducing signal that could cause premature death of Ad-infected cells [156]. Furthermore, parylation requires utilization of the NAD^+^ substrate and, therefore, excessive parylation may cause depletion of NAD^+^ as well as of its precursor, ATP. Such depletion may cause necrotic cell death [157]. Thus, preventing excessive PARP activity in Ad-infected cells contributes to inhibition of various DDR pathways, prevents premature cell death, and averts inhibition of ATP-dependent processes that the virus would require.

## 6. DNA Damage and Oncolytic Ads

Because numerous Ad proteins, and especially E4 proteins, contribute to inhibition of the DDR and, therefore, sensitize cells to the toxic effects of DNA damage, it was suggested that addition of DNA-damaging drugs or irradiation may synergistically augment cancer virotherapy by oncolytic Ads. This hypothesis was confirmed by several studies [158,159,160]. In such studies, utilization of many different Ad vectors in various cell types as well as in tumor xenografts, demonstrated that a combination of Ad infection and DNA damage induced by drugs or irradiation was significantly more toxic than either treatment alone [160,161,162,163]. Moreover, DDR inhibition by drugs also enhanced the impact of oncolytic viruses in tumor cells [158,159]. Infection with Ad vectors did not synergize with DNA damage in normal cells [164]. The combination of enhanced toxicity in cancer cells and minimal toxicity in normal cells is important for cancer therapy.

## 7. Conclusions and Perspectives

The conflict between Ad and the DDR has been investigated for the last twenty five years, providing evidence that although Ad exploits some components of the DDR to improve viral replication, this family of viruses deploys many of its proteins to inhibit DNA damage signaling. These findings indicate that DDR restriction is crucial for Ad replication. Both the incoming genome and newly synthesized genomes are protected from exposure to the DDR via numerous mechanisms. Most of the research in this area focused on interactions between Ads and DDR branches that respond to DSBs and replication stress because Ad replication products, including ds ends and ss replication intermediates, are perceived by the DDR as these types of DNA damage. Interestingly, Ad targets many DNA damage sensors that activate multiple mechanisms, and thus, by directly targeting few key DDR regulators, the virus can efficiently impact several downstream signaling pathways. The understanding of the various mechanisms underlying DDR inhibition by Ad has therapeutic value because it can be utilized when designing cancer virotherapy and anti-virals. Ad proteins that conceal the viral genome from the DDR may also inhibit mechanisms of innate immunity [57,140].

During acute infection with Ad, inhibition of the DDR will not have a lasting effect on the cell, which is destined for lysis. However, persistent infections with Ads have been described, although they are not well understood [165]. If some of the early proteins remain active, they may inhibit the DDR, leading to genome instability in the host cell. This Ad effect in its natural host tissues is still open for investigation.

Altogether, studies of the interactions between Ad and its host cell continue to provide insights into numerous cellular and viral functions.

## Figures and Tables

**Table 1:** Targeting DDR proteins by various orfs of multiple Ad serotypes.

Adenovirus Orfs	MRN	Other DDR-Related Ad Degradation Targets	ATM	ATR	RPA	DNA-PK	DNA-PK-Regulated Pathways (NHEJ)	PARP-1	FANC
E1B-55K	Degradation of Mre11 in collaboration with E4orf6 (Ad4, 5, 12, 40).No Mre11 degradation (Ad3, 7, 9, 11, 34). [88,89]. Degradation of Rad50 or Nbs1 (Ad5, 35). [90,91]. Participation in mislocalization to aggresomes (Ad5). [92,93].	E1B-55k/E4orf6-dependent degradation of p53 (Ad5, 12, 40). Weak or no degradation of p53 (Ad 3, 7, 9, 11, 16, 34). [88,89]. E1B-55k/E4orf6-dependent degradation of Spoc1 (Ad5). Spoc1 restricts Ad5 replication. [87].	Degradation of the ATM activator Tip60 (Ad5). [94]. Degradation of the ATM & ATR substrate TNKS1BP1 (Ad5, 12). No degradation by Ad4, 7, 9, 11. [95].		SMARCAL1 is recruited to Ad RCs in a RPA-dependent manner and its degradation is mediated by E1B-55K/E4orf6 (Ad5, 12). [96].		Degradation of DNA ligase IV, leading to inhibition of genome concatenation by all Ad groups tested [88,89]. Degradation of Blm helicase. Blm knockdown does not affect Ad replication possibly due to redundancy with other helicases (Ad5). [97].	Inhibition of cell death mediated via PARP activation by Ad5. [98].	
E4orf3	MRN mislocalization to E4orf3 tracks (Ad2, 5). No mislocalization (Ad3, 7, 11,12, 35). Inconsistent mislocalization results for Ad4, 9. [64,68,89,99]. Sumoylation of Mre11&Nbs1 at tracks (Ad2, 5). [99]. Mislocalization to aggresomes (Ad5). [92,93].	Epigenetic silencing of p53 target genes. [100].		MRN mislocalization leads to ATR inhibition (Ad5). [60,61].		DNA-PK binding and inhibition of genome concatenation (Ad5). [37,101].		Inhibition of cell death mediated via PARP activation by Ad5: same as E1B-55K. [98].	
E4orf4			ATR-independent, DNA-PK-dependent inhibition of ATM (Ad5). PARP inhibition enhances E4orf4-induced ATM attenuation. [102].	ATM-independent, DNA-PK- and PP2A-dependent inhibition of ATR (Ad5). PARP inhibition enhances E4orf4-induced ATR attenuation. [102].		DNA-PK binding. Early activation and late inhibition of DNA-PK autophosphorylation. DNA-PK inhibition at late times improved Ad5E4Δ mutant replication better than early inhibition (Ad5). [62].		PARP-1 binding. PP2A-dependent inhibition of parylation induced by DNA damage or Ad infection (Ad5). PARP inhibition enhanced replication of an Ad5E4Δ mutant. [103].	
E4orf6	Degradation of MRN components in collaboration with E1B-55K (see E1B-55K above).	Degradation of p53 and Spoc1 in collaboration with E1B-55K (see E1B-55K above).	Degradation of Tip60 and TNKS1BP1 in collaboration with E1B-55K (see E1B-55K above).	Degradation of the ATR activator TOPBP1 without involvement of E1B-55K (Ad12 only). [104].	Degradation of SMARCAL1 in collaboration with E1B-55K (see E1B-55K above).	DNA-PK binding. Inhibition of V(D)J recombination (regulated by DNA-PK). Inhibition of genome concatenation (Ad5). [37,101].	Degradation of DNA ligase IV and Blm helicase in collaboration with E1B-55K (see E1B-55K above).		
pVII			SET/TAF-1-mediated prevention of ATM activation by the incoming Ad5 genome which is coated by pVII. [79,81].						
Whole virus information	Validated inhibition of Ad replication by MRN (Ad9, 12, Ad5E4Δ). Activation (Ad35). [68,71,105].		ATM activation in RCs of Ad2, 4, 9, 12, 35. [68]. ATM suppression at RCs by Ad5. [45,66]. Global ATM activation by Ad3, 4, 5, 7, 9, 11, 12. [45,89]. ATM did not impair replication of Ad9, 12. [68]. ATM inhibition enhanced Ad5E4Δ mutant replication but reduced Ad12 replication. [45,68,102].	Constant activation of ATR determined by pChk1 levels (Ad4, 11, 7). Transient activation (Ad3). Suppression of ATR activation (Ad5, 9, 12). [89].	Ad12 induces RPA32 phosphorylation. [59].	Early but not late colocalization of DNA-PK with Ad5 RCs. [62].		PARP-1 is recruited to Ad5 RCs. [103]. Ad5 replication stimulates cellular and viral protein parylation. [98,103].	Ad5 induces the FANC pathway resulting in enhanced virus replication. [69].
